# Supplementary material for: Functional implication of the homotrimeric multidomain vacuolar sorting receptor 1 (VSR1) from Arabidopsis thaliana
Source: Sci Rep. 2024 Apr 26;14:9622. doi: 10.1038/s41598-024-57975-2 (PMC11052993; doi:10.1038/s41598-024-57975-2)
Supplement: Supplementary file 1 — Supplementary Information. [file 41598_2024_57975_MOESM1_ESM.pdf]

Suppl. Table 1

	Residue EGF-like (4 residues)	Distance (Å)	Residue in TRX (4 residues)	H-bond characteristics
1	GLU 414 [OE2]	2.6	LYS 221 [NZ]	Salt
2	ASN 418 [ND2]	3.34	SER 326 [O]	
3	ARG 440 [NH2]	2.68	ASP 303 [OD1]	Salt
4	ARG 440 [NH2]	2.74	ASP 303 [OD2]	Salt
5	ARG 440 [NH1]	3.34	ASP 303 [OD2]	Salt
6	ARG 440 [O]	2.92	LYS 213 [NZ]	
7	TYR 457 [OH]	2.72	ASP 303 [OD1]	

Suppl. Table 2

**Wavelength (Å)**	1.0108
**Resolution range (Å)**	36.39 - 3.5 (3.57 - 3.5)
**Space group**	P 2_1 3
**Unit cell (Å and °)**	a= 145.543, b= 145.543, c= 145.543, a= 90, b=90, g=90
**Completeness (%)**	96.28 (87.07)
**Mean I/sigma(I)**	21.72 (3.86)
**Wilson B-factor (Å^2)**	67.05
**R-merge**	0.03321 (0.1491)
**CC1/2**	0.997 (0.909)
**Reflections used in refinement**	24159 (1125)
**Reflections used for R-free**	1299 (114)
**R-work**	0.1803 (0.2464)
**R-free**	0.2160 (0.2730)
**CC(work)**	0.891 (0.774)
**CC(free)**	0.869 (0.749)
**Protein residues**	427
**RMS(bonds)**	0.006
**RMS(angles)**	0.870
**Ramachandran favored (%)**	95.98
**Ramachandran allowed (%)**	3.55
**Ramachandran outliers (%)**	0.47
**Rotamer outliers (%)**	0.00
**Clashscore**	8.95

Suppl. Information

Full sequence of crystallized protein. The full sequence of VSR1 construct that was used for crystallization is shown below. The blue letters represent the signaling peptide that was processed during the expression, and the red letters represent the C-terminal tag including the 6XHis-tag. The black bold letters represent residues between 20 and 560 of AtVSR1.

MKLGLFTLSFLLILNLAMG**RFVVEKNNLKVTSPDSIKGIYECAIGNFGVPQYGGTLVGTVVYPKSNQKACKSYSDFDISFKSKPGR**
LPTFVLIDRGDCYFTLKAWIAQQAGAAAILVADSKAEPLITMDTPEEDKSDADYLQNITIPSALITKTLGDSIKSALSGGDMVNMK
LDWTESVPHPDERVEYELWTNSNDECGKKCDTQIEFLKNFKGAAQILEKGGHTQFTPHYITWYCPEAFTLSKQCKSQCINHGRYCA
PDPEQDFTKGYDGKDVVVQNLRQACVYRVMNDTGKPWVWWDYVTDFAIRCPMKEKKYTKECADGIIKSLGIDLKKVDKCIGDPEAD
VENPVLKAEQESQIGKGSRGDVTILPTLVVNNRQYRGKLEKGAVLKAMCSGFQESTEPAICLTEDLETNECLENNGGCWQDKAANI
TACRDTFRGRLCECPTVQGVKFVGDGYTHCKASGALHCGINNGGCWRESRGGFTYSACVDDHSKDCKCPLGFKGDGVKNCEDVDEC
KEKTVCQCPECKCKNTWGSYECSCSNGLLYMREHDTCIGSGKVGSGGLESRGPFEGKPIPNPLLGLDSTGTGHHHHHH

Suppl. Fig. 1

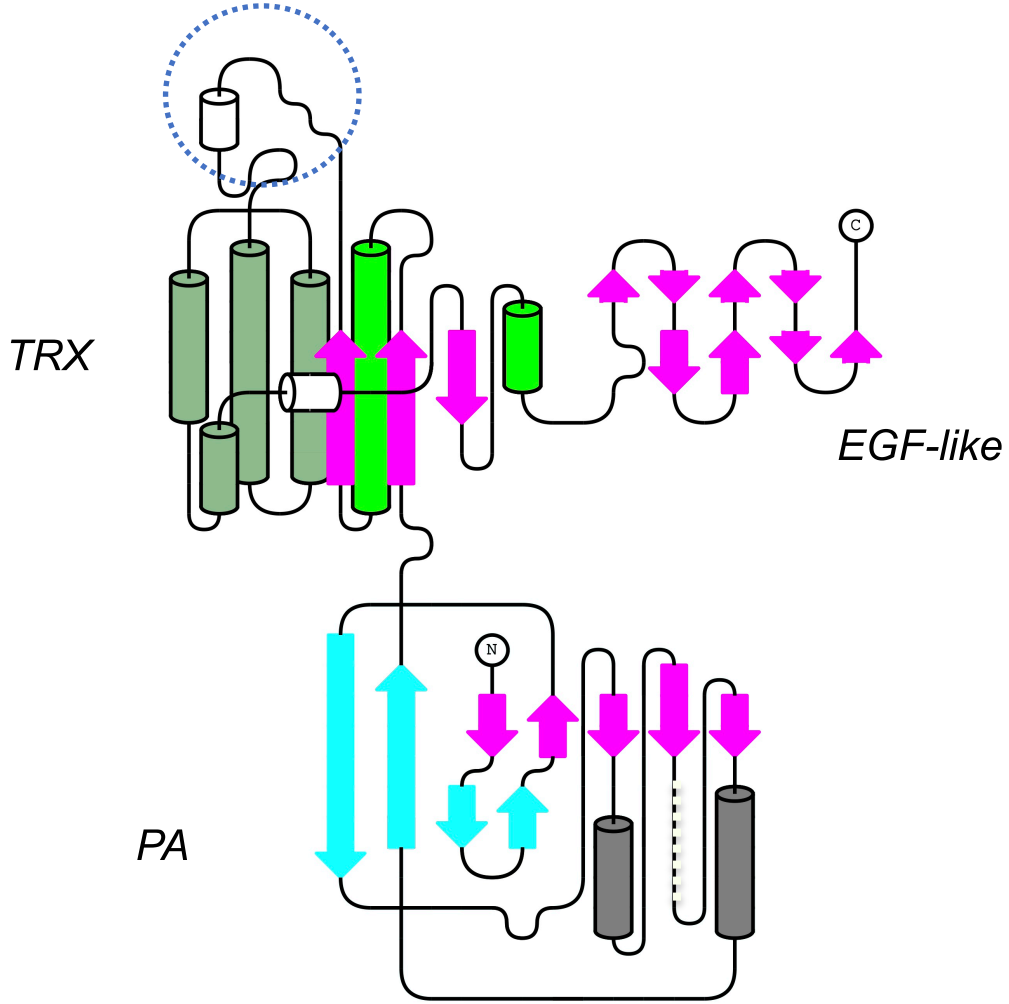

Suppl. Fig. 1 The overall topology of AtVSR1. Each domain is labeled. The PA domain features a central β-barrel composed of two β-sheets colored in cyan and magenta. A dashed line indicates a disordered region. The TRX domain consists of a canonical βαβ motif and a βα motif in magenta/green, separated by three structural components; a long insert marked by a dashed circle, an α-helical bundle in pale green and an α-helix in white.

Suppl. Fig. 2

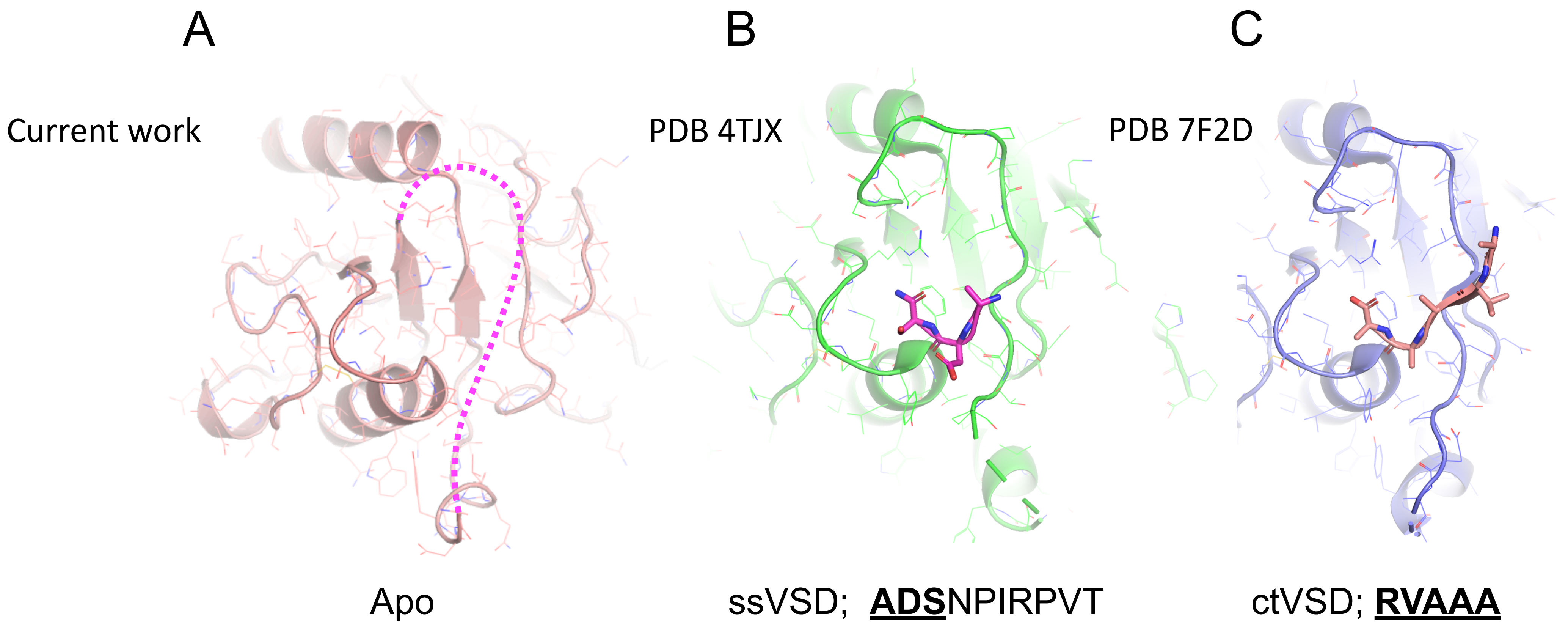

Suppl. Fig. 2 Comparison of three crystal structures of AtVSR1 PA domains. A, The magenta dashed line in the Apo structure represents the disordered loop residues. B and C, the same loop is ordered and is involved in peptide ligand bindings. The peptide sequences used for crystallizations are shown below each structure. The bold underlined sequences are resolved in the structure.

Suppl. Fig. 3

Suppl. Fig 3. AF2 model of AtVSR1 (accession number; AF-93026-F1). A, AF2 prediction of AtVSR1. B and C, The AF2 model and the model based on the crystal structures are aligned via the PA domains (B) and TRX domains (C), respectively. The comparison of the domain interface with that of the crystal structure shows that the AF2 prediction differs in domain interfaces. Note that the first EGF-like domain is not interacting with the TRX domain, and the rest of the EGF-like domains are in an extended conformation in the AF2 model. RMSD values comparing the AF2 model and the crystal structure for each domain are shown. The PA, TRX, and EGF-like domains are colored salmon, green, and blue, respectively. The rest of the model not resolved by the crystal structure is colored yellow which includes two EGF-like domains, a linker, the transmembrane domain, and the C-terminal cytosolic residues.

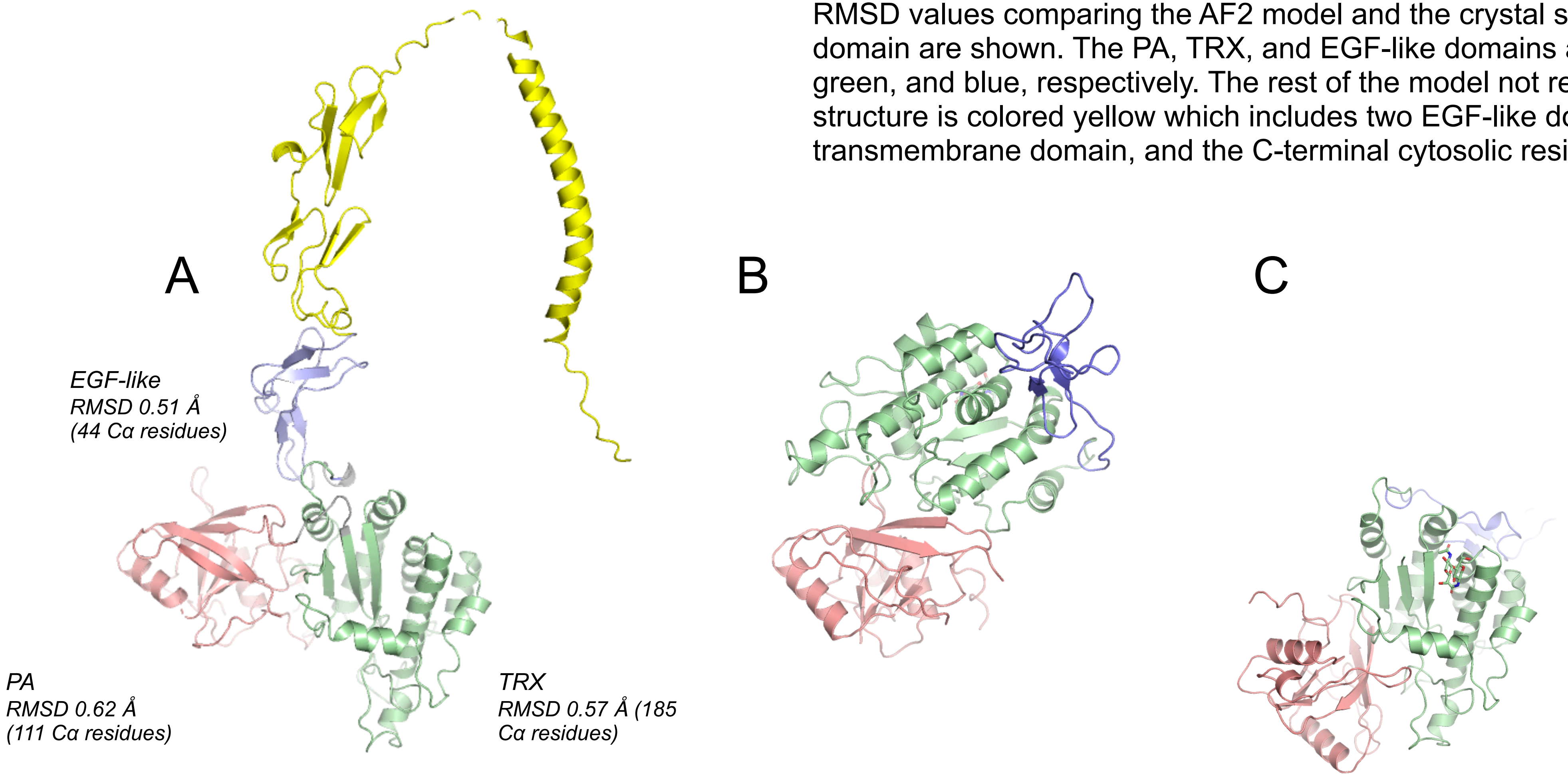

Suppl. Fig. 4

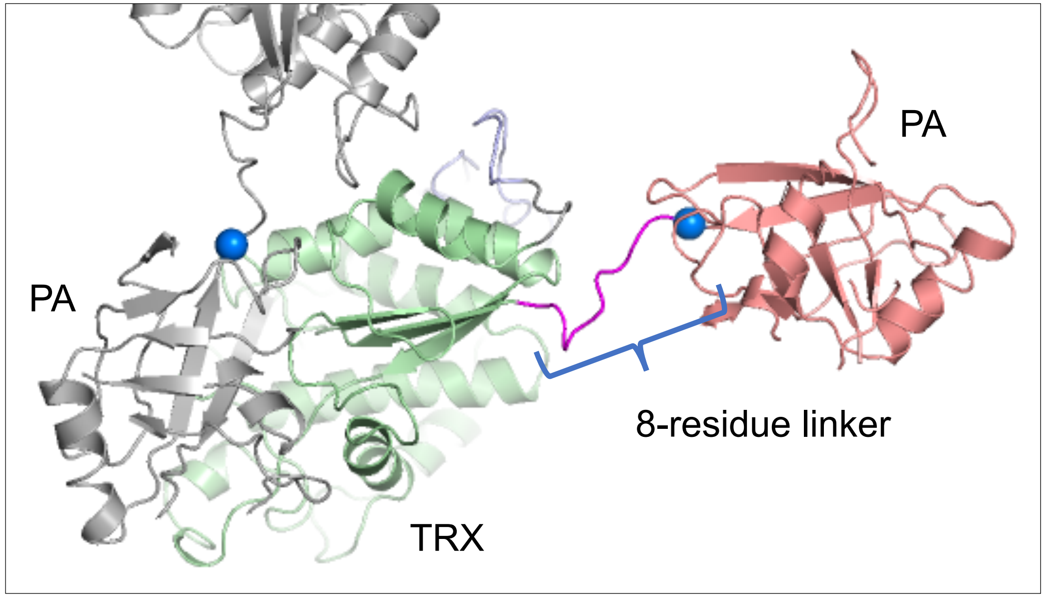

Suppl. Fig.4. The linker residues between the PA and TRX domains. The blue spheres (representing the Cα of Thr176) indicate the same positions in two different chains, and the two spheres should overlap for interdomain interaction within the same molecule to occur. The linker residues are colored magenta. The asymmetric molecule is colored gray.

Suppl. Fig. 5

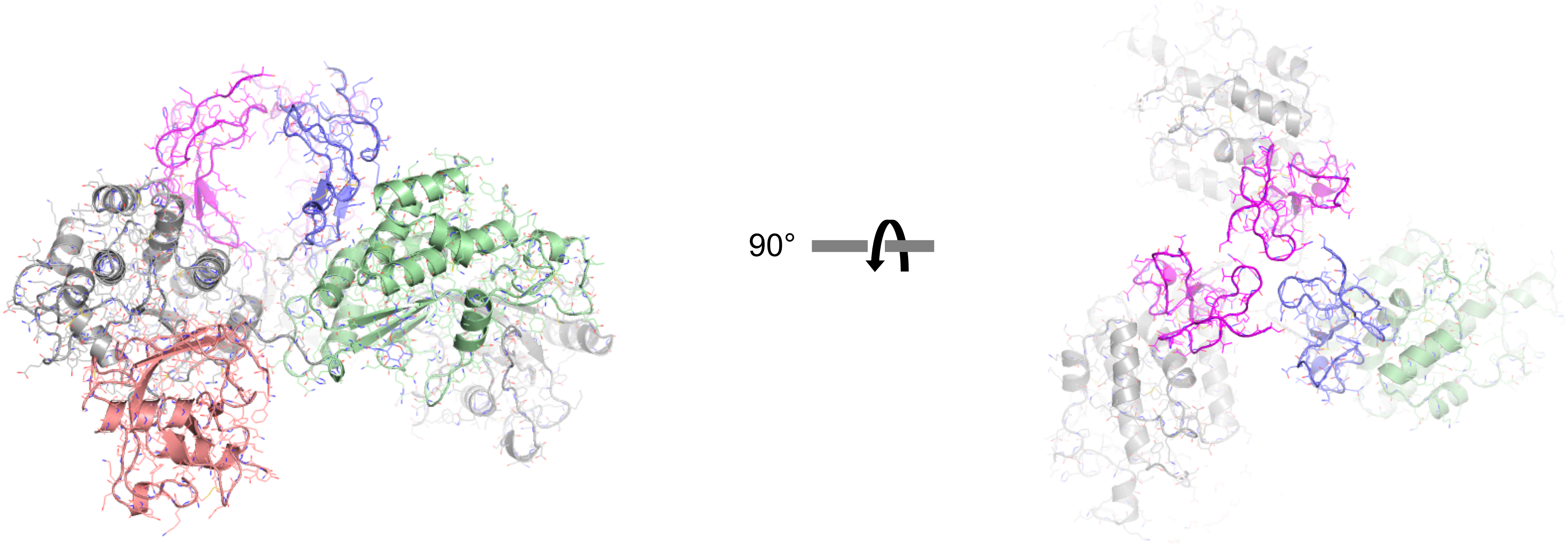

Suppl. Fig. 5. Crystallographic 3-fold symmetry of AtVSR1 showing minimal interaction among the EGF-like domains. The PA, TRX, and EGF-like domains are colored salmon, green, and blue, respectively. Two other symmetry-related molecules are colored gray, except for the EGF-like domains, which are colored magenta for clarity.

Suppl. Fig. 6

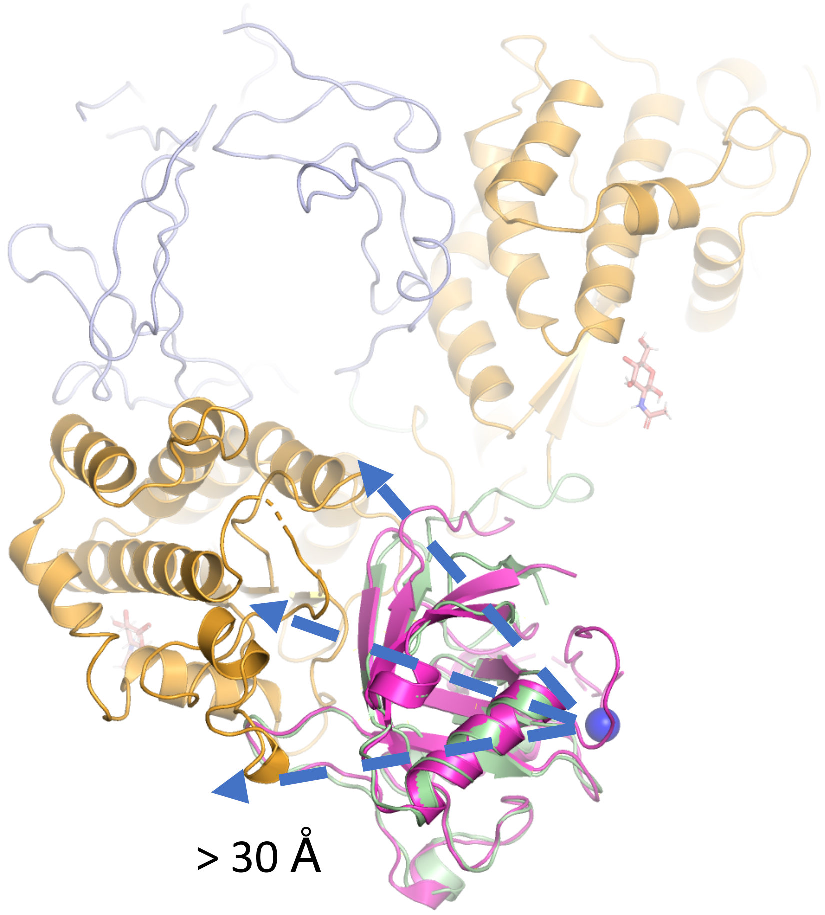

>Arabidopsis thaliana Aleurain

MSAKTILSSV VLVVLVAASA AANIGF<u>**DES**</u>N PIRMVSDGLR…. .

Signaling peptide

NPIR motif

Suppl. Fig. 6. The structure of the PA domain-Aleurain peptide complex (PDB ID 4TJX) was superimposed onto the PA domain of our crystal structure. The closest distance between the aleurain binding site of the PA domain and the TRX domain, where the NPIR motif is predicted to bind, is at least 30 Å apart. The underlined sequences are resolved in the PDB 4TJX. The PA and TRX domains are colored green and orange, respectively. The PDB ID 4TJX is colored magenta, and the blue sphere represents the peptide binding site.

Suppl. Fig. 7

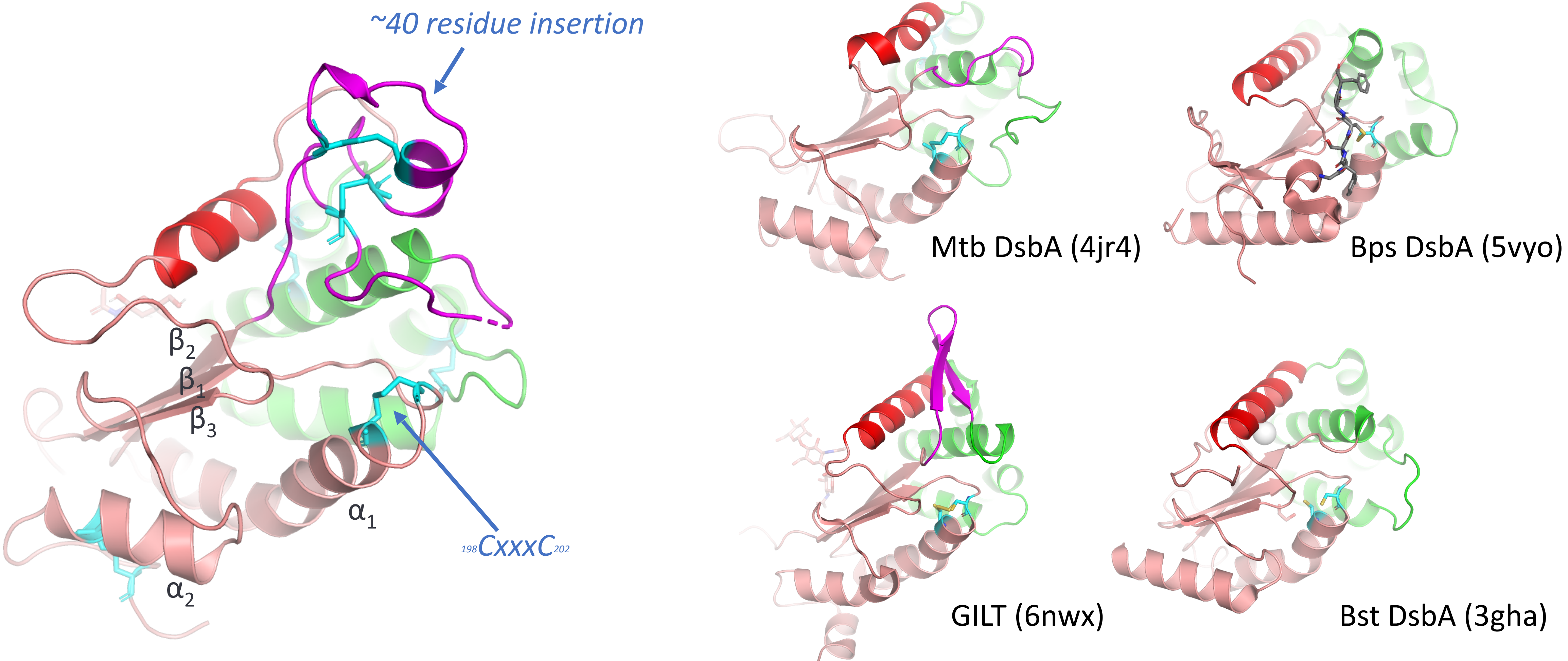

Supp. Fig. 7. The folding of the AtVSR1 TRX domain is shown. The core TRX topology ($\beta_1\alpha_1\beta_2$ - $\beta_3\alpha_2$) is colored salmon, and an α-helical insertion between $\beta_1\alpha_1\beta_2$ and $\beta_3\alpha_2$ is colored red. An α-helical bundle is colored green. An insertion loop with α-helix and two disulfide bonds located between the TRX fold and the helical bundle is colored magenta. Additional disulfide bonds, including the CxxxC motif, are shown in cyan. The top-scoring structures from the DALI search are included for comparison. The coloring schemes used for all structures are identical.

Suppl. Fig. 8

A

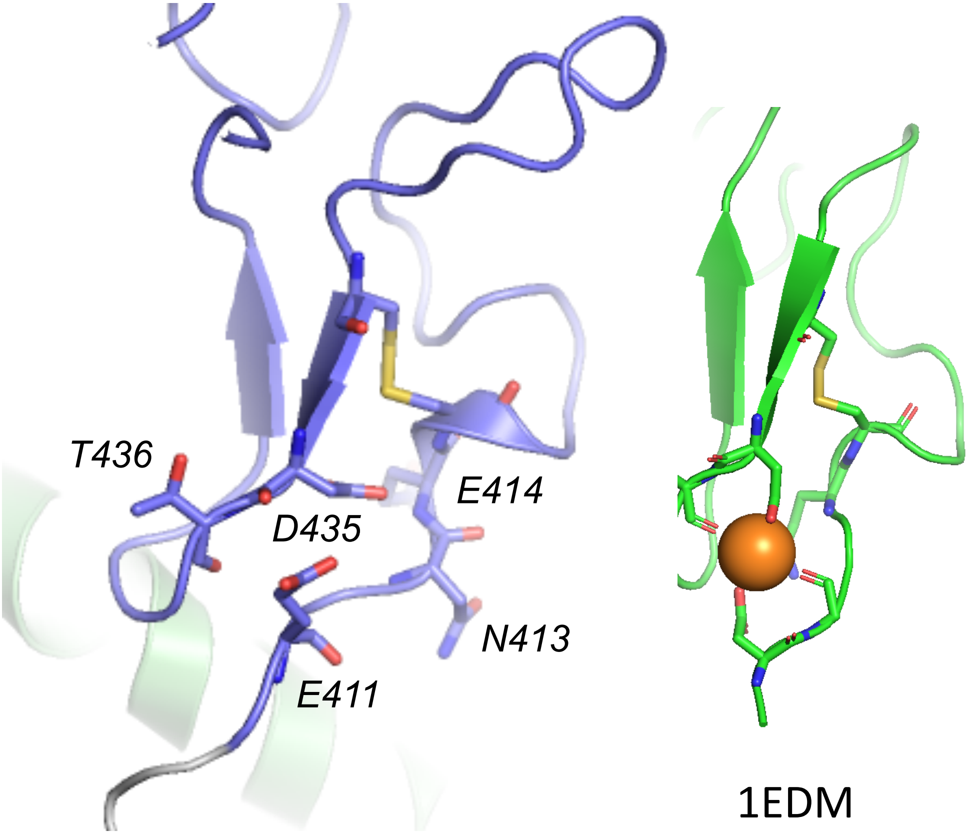

B

The first EGF-like domain (411-461)

ETNECLENNGGCWQDKAANITACRDTFRGRLCECPTVQGVKFVGDGYTHCK

The second EGF-like domain (464-511)

GALHCGINNGGCWRESRGGFTYSACVDDHSKDCKCPLGFKGDGVKNCE

The third EGF-like domain (512-554)

DVDECKEKTVCQCPECKCKNTWGSYECSCSNGLLYMREHDTCIGS

Supplementary Figure 8. Sequence conservation of EGF-like domains in AtVSR1. A. The crystal structure of the AtVSR1 EGF-like domain was compared to epidermal growth factor (PDB ID 1EDM). The residues that are conserved for Ca^{2+} binding are shown as sticks. **B**. The sequence comparison of EGF-like domains in AtVSR1 is shown. The brackets above Cys residues indicate disulfide bond pairs. The underlined residues are predicted to be involved in Ca^{2+} binding, and the cyan color represents the conserved residues. The third EGF-like domain has an additional disulfide bond, as indicated by an AF2 full-length model (AFP93026F1).
